# Detrending Changes the Temporal Dynamics of a Semantic Fluency Task

**DOI:** 10.3389/fnagi.2016.00252

**Published:** 2016-10-25

**Authors:** Steven Lenio, Frances M. Lissemore, Martha Sajatovic, Kathleen A. Smyth, Curtis Tatsuoka, Wojbor A. Woyczynski, Alan J. Lerner

**Affiliations:** ^1^School of Medicine, Case Western Reserve UniversityCleveland, OH, USA; ^2^Departments of Psychiatry and Neurology, University Hospitals Cleveland Medical CenterCleveland, OH, USA; ^3^Department of Mathematics, Applied Mathematics and Statistics, and Center for Stochastic and Chaotic Processes in Science and Technology, Case Western Reserve UniversityCleveland, OH, USA

**Keywords:** semantic fluency testing, semantic memory, cognitive impairment, Alzheimer’s disease, Weibull distribution, cluster-switch analysis

## Abstract

**Objective**: To study the dynamics of clustering semantic fluency responses and switching between clusters.

**Methods**: We conducted a cross-sectional study of participants (*N* = 60) in a study of patient reported outcomes who were given the Saint Louis University Mental Status test. Sixty-second animal naming tests were scored for the timing of responses as well as the clustering of responses into semantic categories. Time scores were detrended to correct for exponential exhaustion and normalize the time scale across individuals.

**Results**: Grouped by number of responses given, low performers (LP; Carter et al., 2012) switched between clusters fewer times than medium performers (MP) and high performers (HP). Prior to detrending, LP showed increased intracluster response times when compared to the other groups, but no differences were shown in intercluster response times. After detrending, however, the difference in intracluster response times disappeared and LP showed significantly faster detrended intercluster response times compared to both MP and HP.

**Conclusion**: Prior to detrending, slower intracluster response times appear to be driving poorer performance. When time scores are detrended, our findings suggest that LP participants have quicker intercluster response times but exhaust more quickly as well. Detrending can help describe the interplay between the structure-loss and retrieval-slowing models of declining semantic fluency by isolating the component mechanisms involved in each.

## Introduction

Category fluency testing, a method of testing semantic memory, is sensitive to early cognitive changes in Mild Cognitive Impairment (MCI) and Alzheimer’s disease (AD), and has also been useful in studying general mechanisms underlying semantic memory recall (Kim et al., 2011; Eastman et al., 2013; Hirni et al., 2013; Roca et al., 2013; Sung et al., 2013). A commonly used measure of semantic fluency, the animal naming test, requires participants to name as many animals as possible over a given time period, generally 60 s. Diagnostic neuropsychological testing has generally focused solely on the total number of animals named (N60); however, several studies have examined the patterns and dynamics of the participants’ responses in an attempt to better understand the mechanisms underlying semantic recall and how these mechanisms are disrupted with cognitive impairment (Rohrer et al., 1995; Troyer et al., 1997; Lerner et al., 2009; Meyer et al., 2012; Bertola et al., 2014a,b; Weakley and Schmitter-Edgecombe, 2014).

Two frequently proposed models for the decreased semantic fluency observed with cognitive impairment are the structure loss model and the retrieval-slowing model. The structure loss model attributes word-finding deficits to a breakdown in the associative networks underlying semantic memory. The retrieval-slowing model attributes these deficits to a more widespread, general slowing of retrieval processes while the underlying semantic structure remains intact (see Rohrer et al., 1995 for a good discussion of these models).

Rohrer et al. (1995) developed an exponential model to describe the decreased production of responses over time, suggesting that the rate of decline corresponds to the variability in speeds of recall processes. In our own work (Meyer et al., 2012), we termed this decline exponential exhaustion, and described a method called detrending that normalizes individual variability in rates of recall across the test epoch.

Detrending is a “…non-linear procedure (that) permits us to see the local structure of the intercall times independently of the individuals’ exponential exhaustion rates” (Meyer et al., 2012). Intercall time is the duration between responses during the animal naming task. Detrending produces a new, unitless time score such that the responses occur at a rate of one response per unit time, yielding a linear sequence of responses in place of an exponential curve. As a result, the detrended intercall times can now be considered random quantities with a similar probability distribution and can be used to derive the common underlying statistical characteristics of the intercall times. Detrended intercall times from individuals in the same performance group can thus be pooled to produce larger sample sizes, assuring tighter confidence intervals for the statistical parameters to be estimated. Having controlled for the varying speeds of retrieval across individuals, these common statistical characteristics can shed light on the structure of the semantic memory retrieval process.

By comparing the data prior to detrending (termed raw data here) with the detrended results, we can separate effects attributable to differences in retrieval speed from those that reflect the underlying structure and process of semantic retrieval. With the structure loss hypothesis we would expect the semantic deficits to be reflected as slower intracluster response times, while intercluster times would be relatively unaffected. If general retrieval slowing drives semantic deficits, then we would expect global slowing in both intra- and intercluster response times. Since detrending removes the effects of retrieval slowing, any changes between raw data analysis and detrended analysis must then reflect an influence of retrieval slowing. Further, if differences in clustering patterns among groups are still apparent after detrending, then additional factors that affect semantic recall are implicated.

The Weibull distribution approximates the distribution of raw and detrended intercall times, and we can employ its associated variables to dissect the response patterns observed across the study population. The Weibull variables of particular interest here are *β*, the shape constant, *N*_∞_, the theoretical asymptotic upper limit of how many words can be produced that is derived from N60, the total number of responses by the participant, and *τ* (tau), the (inverse) rate at which that limit is achieved (see Meyer et al., 2012 for further discussion of the Weibull probability function and variables).

Troyer et al. (1997) first described the processes of clustering and switching whereby semantically associated responses are clustered with distinct switches observed between sequential clusters. They compared the clustering and switching trends of younger vs. older adults, while more recently others have compared the clustering and switching trends across groups with varying cognitive impairment (Sung et al., 2013; Weakley and Schmitter-Edgecombe, 2014). The current study, a secondary analysis from a larger longitudinal study in elderly individuals at risk for dementia, assessed performance on the animal naming portion of the St. Louis University Mental Status (SLUMS) test (Tariq et al., 2006). The aim was to identify category clustering and switching trends after removing the confounding effects of exponential exhaustion in order to shed light on the underlying semantic processes that occur in neurodegenerative disorders.

## Materials and Methods

### Subjects

Participants (*N* = 60) were drawn from a larger study of patient reported outcome measures in older adults (“Assessing Early Alzheimer and At-Risk Groups with Patient Reported Outcomes”). All participants signed an informed consent document approved by the University Hospitals of Cleveland Case Medical Center IRB. If participants with cognitive impairment were unable to summarize the study procedures after undergoing the consent process, a legally authorized representative also signed the consent form. Participants were recruited to the larger study based on the following criteria:

Inclusion Criteria
Age 70 years or older; Mini–Mental State Examination (MMSE) score of 16 or higher; able to read and speak English; and able to provide informed consent at the time of the initial baseline interview.

Exclusion Criteria
Life expectancy less than 12 months; planned nursing home placement or move from the study area within the upcoming 12 months; active substance abuse or dependance; and severe, uncontrolled mental disorder that would render the individual unable to complete a questionnaire.

Participants were assessed twice, at baseline and 12 months later. Participants were assessed with a battery of self-report and rater administered scales. Diagnostic work-ups were not performed. The category fluency testing reported here was administered at 12 months.

### Category Fluency Testing

The SLUMS, which contains a 60-s animal naming test, was administered to all participants at baseline and at 12 months. Cognitive group assignment for the larger study was determined using SLUMS scores and educational attainment as follows: for those with high school education or greater, SLUMS cutoff scores were normal, MCI and dementia were 27–30, 21–26, and 1–20, respectively. For those with less than High school graduation, the cutoffs were 25–30, 20–24, and 1–19 (Tatsuoka et al., 2016). These cutoffs have been found to be highly sensitive in both high and low educational attainment groups (Tariq et al., 2006). At 12 months, participants with baseline scores indicating normal cognition, MCI, and dementia were asked to permit audio recording of the animal naming portion of the SLUMS until 20 from each group agreed. We re-assigned the groups to emphasize performance outcome on the verbal fluency task: those results showed a break in the distribution of the number of responses between 11 and 13 (i.e., no one produced 12 words), suggesting a natural divide between low performing and medium performing participants; we chose a cutoff between 18 and 19 responses to distinguish medium and high performing participants. This breakdown produced tertiles based solely on animal naming task scores: low performers (LP, *n* = 20) produced 11 or fewer words; medium performers (MP, *n* = 21) produced 13–18 words; and high performers (HP, *n* = 19) produced more than 18 words (These group sizes roughly equaled those attained by the SLUMS score cognitive ability breakdown).

The recordings were transcribed noting the time of word onset using WavePad Sound Editor (NCH Software, Inc., Greenwood Village, CO, USA). Two raters scored the sequences of animal names for clusters and switches according to the methods outlined by Troyer et al. (1997), with one difference: we assigned individual responses that were not clustered a cluster size of 1 (instead of 0, as per Troyer’s methods). Clusters consisted of animals named sequentially that belong to the same zoological category, natural environment, or human use category. We measured mean cluster size and number of switches between clusters. Intercall times—the time from the start of one response to the start of the next response—were grouped into intracluster response times (time between responses within a cluster) and inter-cluster response times (time between last response of a cluster and the first response of the next cluster). We counted only unique responses and no non-animal responses were made.

Occasionally, an animal name was encountered that bridged two clusters and could reasonably be assigned to either cluster. For example, if a subject said “… rabbit, dog, cat, lion, tiger, leopard…”, “cat” belongs to a human use category (pets), as well as a zoological category (felines). In such a circumstance, “cat” belongs to both clusters, and the responses would be scored as two clusters of 3 (rabbit, dog, cat) and 4 (cat, lion, tiger, leopard), respectively. Intracluster response times would be calculated between dog and cat as well as cat and lion. When considering the intercluster response time between these clusters, we would average the response times between dog and cat and between cat and lion to account for the bridging effect that “cat” has between the two categories.

The Weibull distribution was calculated for the detrended data using methods described in Meyer et al. (2012).

### Statistical Analyses

Intercall time, intracluster time and intercluster time results were analyzed using one-way analysis of variance (ANOVA) with Tukey’s multiple comparisons post-test or unpaired *t*-tests where applicable with Prism 6 software (GraphPad Prism, Inc., LaJolla, CA, USA).

## Results

Comparisons among the groups showed a significant difference in age between HP and LP (*p* < 0.005), and the HP group had more years of education than LP (*p* < 0.01). The mean SLUMS score was lower for LP than for MP and for HP (both comparisons *p* < 0.001; Table 1).

**Table 1:** **Demographics**.

	Low performers	Medium performers	High performers
Age	81.2 (6.2)*	78.8 (5.3)	75.3 (5.5)
Education	2.5 (1.43)	3.0 (1.20)	3.79 (1.15)^§^
SLUMS score	13.95 (4.4)^‡^	22.81 (4.9)	25.68 (3.5)

All values shown are mean (sd). Pair-wise comparisons: *LP older than HP, p < 0.005; Education code: 1–5, 1 = did not finish high school, 5 = post-graduate. ^§^HP more education than LP, p < 0.01. ^‡^LP lower than MP and HP, both comparisons p < 0.001. All other comparisons p > 0.05.

**Figure 1:**
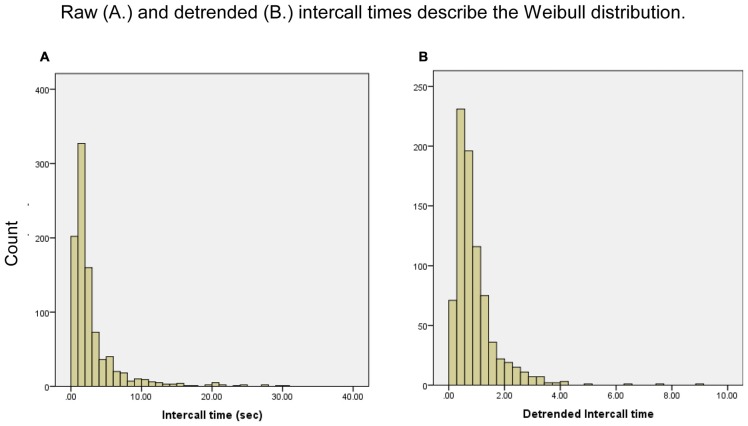
**Frequency distribution of intercall times, raw and detrended.** Frequencies are for responses anywhere in response sequences; for example, the first bar in **(A)** represents all the intercall times of less than 1 s regardless of where they occurred in all participants’ sequences, from a first response to a 37th response.

While the distribution of the raw and detrended intercall times closely match the Weibull distribution (Figure 1), few of the Weibull parameters varied significantly among the groups. As expected, the significant differences in total response production (N60) among the groups were also reflected in *N*_∞_ (N60: HP produced more words than LP or MP, both *p* < 0.005; MP produced more than LP, *p* < 0.001; *N*_∞_: HP greater than LP and MP, both comparisons *p* < 0.001), and τ differed significantly between LP and HP (*p* < 0.05). *β* did not distinguish among the three groups of participants (Tables 2A,B).

**Table 2:** **Weibull variable values and comparisons**.

	A. Weibull variable values (Mean sd)			B. Weibull pair-wise comparisons		
	Low performers	Medium performers	High performers	LP–MP	LP–HP	MP–HP
N60	8.3(2.1)	15.6(1.7)	23.8(4.8)	*	**	**
N_∞_	13.40(30.2)	26.71(13.65)	50.41(19.89)	ns	^‡^^‡^	^‡^^‡^
τ	36.6(0.1)	52.71(41.51)	72.02(43.29)	ns	^‡^	ns
γ	0.2(0.5)	0.29(0.12)	0.27(0.09)	ns	ns	ns
β	1.1(0.2)	1.06(0.34)	1.11(0.19)	ns	ns	ns
η	0.7(0.2)	0.69(0.19)	0.76(0.11)	ns	ns	ns

*MP more words than LP, p < 0.001, **HP more words than MP or LP, both p < 0.005. ^‡^^‡^HP greater than LP and MP, both p < 0.001; ^‡^HP greater than LP, p < 0.05.

**Figure 2:**
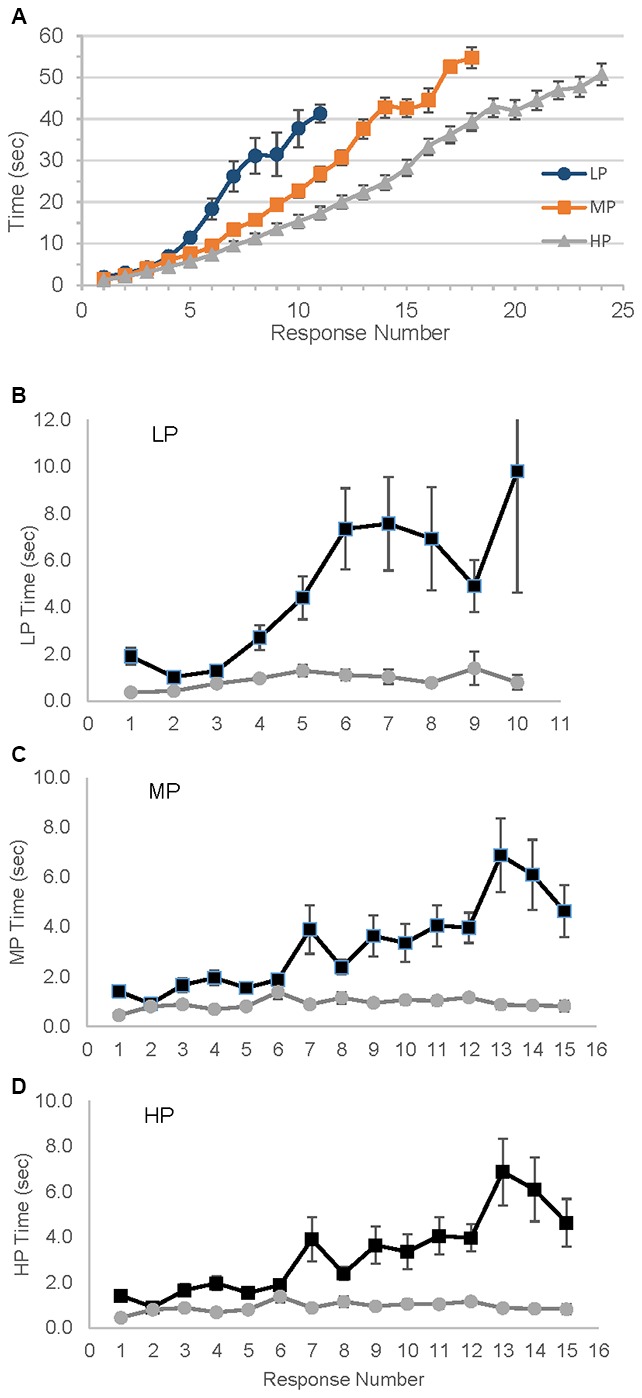
**Variability in time needed to produce number of responses.** LP, Low performer; MP, Medium performer; HP, High performer. **(A)** Mean time in seconds to reach each response; error bars represent standard error. **(B–D)** Mean Intercall times, raw and detrended times for each group. Detrended time is unitless. ▀ Raw data, 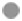 Detrended data.

The differences among the groups in rate of response production were striking, demonstrating the need for the detrending procedure that normalizes these dramatic differences (Figure 2A). For each group the mean amount of time to produce responses as the animal naming test progressed are shown, and after only 5 s and three responses the groups began to diverge in rate of production. Figures 2B–D show the results after detrending.

**Figure 3:**
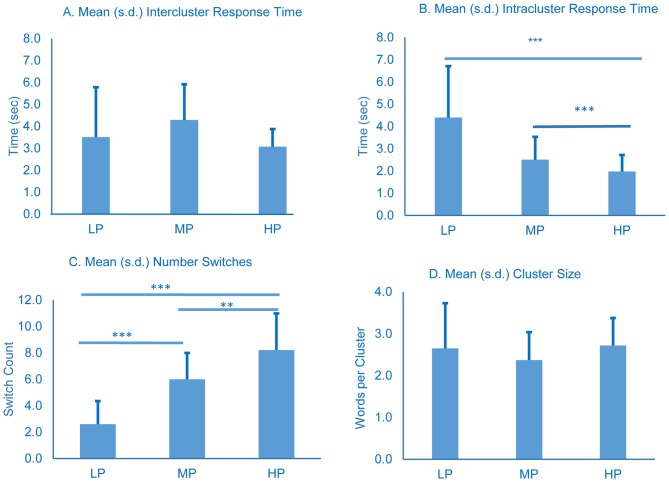
**Cluster and switch analysis for all groups.** All time in seconds. Error bars represent standard deviation. *p*-values were determined using one-way ANOVA with Tukey’s post-test; **p < 0.01; ***p < 0.001.

Switches were scored by two raters. Spearman correlation coefficient for inter-rater reliability was 0.89 and one rater’s scoring was used in the analysis. No significant differences were observed in mean intercluster response times (Figure 3A). LP spent significantly longer between responses within a cluster (intracluster time) compared to the other groups, and MP spent longer than HP (all comparisons *p* < 0.001, Figure 3B). All three groups differed significantly from each other in number of switches (see figure for *p*-vaules, Figure 3C), and mean cluster size did not vary significantly among the groups (Figure 3D).

**Figure 4:**
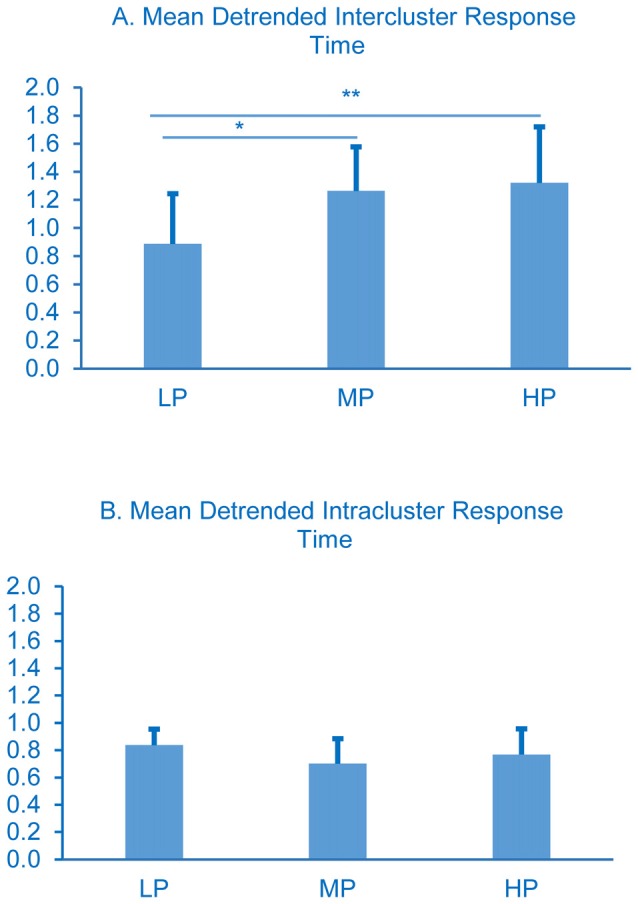
**Detrended cluster switch characteristics.** Intercluster comparisons LP less than MP and HP, *p < 0.05 and **p < 0.01 respectively. All intracluster comparisons *p* > 0.05. Error bars represent standard deviations, *p*-values were determined using one-way ANOVA with Tukey’s post-test.

The detrended data show a different pattern. Detrended intercluster response times varied significantly; LP spent less time between clusters (Figure 4A) than MP (*p* < 0.01) or HP (*p* < 0.001), but the intracluster response times did not (Figure 4B).

A similar analysis on these data using the SLUMS score cutoffs for cognitively normal, MCI and dementia (data not shown) yielded similar results to those above.

**Figure 5:**
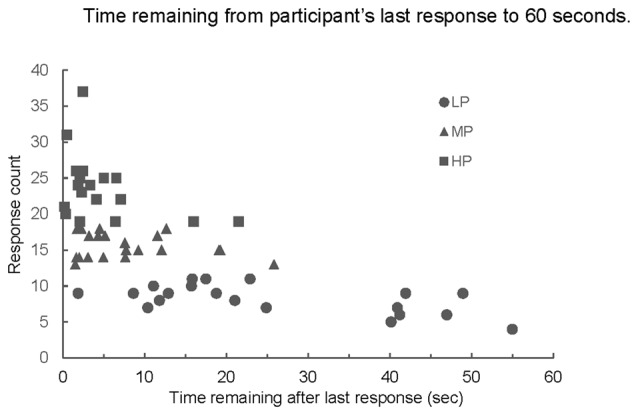
**Number of responses produced by time remaining from last response to end of 60-s trial.** Mean time remaining for LP was greater than mean time remaining for MP or HP (both comparisons *p* < 0.001); MP and HP not significantly different (*p* > 0.49).

An interesting phenomenon appeared in the response sequences. All participants were given 60 s to complete the task, but the average amount of time between the last response given and the stop point at 60 s varied significantly among the groups (*p* < 0.001, Figure 5). LP averaged more time remaining than both MP and HP (both comparisons; *p* < 0.001, Figure 5); time remaining for MP and HP did not differ significantly from each other.

## Discussion

This analysis of category fluency in a sample of older adults using the animal-naming portion of the SLUMS suggests that recall patterns might be explained by multiple models of semantic memory decline. Our findings demonstrate that rates of exponential exhaustion vary widely in semantic fluency testing, and detrending the raw data strongly influences the patterns of intra- and intercluster response times. The distribution of our data clearly demonstrates that intercall times of the semantic fluency task fit the Weibull distribution (Figures 1A,B). Deriving the Weibull variables, of which detrending is a part, allows us to finely parse the factors that affect response production, clarifying which cognitive processes might be involved in producing observed patterns.

In Meyer et al. (2012), we showed that the shape parameter of the Weibull distribution, *β*, could significantly distinguish between the detrended intercall times of younger and older adults. Here, however, where all participants were older adults, *β* made no such distinction among the groups, and only τ the time constant showed any significant difference among the groups (LP lower than HP, Table 2). Lack of variability in the Weibull parameters in this study (in contrast to our prior work with younger and older adults; Meyer et al., 2012) suggests that the Weibull parameters may be better suited to distinguishing age-related effects on semantic fluency than dementia related effects (Buckner, 2004; Haugrud et al., 2011; Nelson et al., 2011; Zamarian et al., 2015; Xu et al., 2016 #173), and that differences are revealed by the detrending process.

Previous studies have suggested that competing models, retrieval slowing and structure loss, underlie decay in semantic recall and that the ability to switch between semantic categories during a semantic fluency test is related to frontal lobe functioning (Troyer et al., 1997; Hickok and Peoppel, 2004). As is typical for the animal naming task, our participants were given 60 s to generate responses. The LP group showed a large range in the amount of time remaining from their last response to the 60-s end point of the trial (Figure 5). On average, LP participants had more than 44 s remaining in the trial after they produced their last response. While the pattern clearly indicates that for this sub-group retrieval is impaired, we propose that the two models are not mutually exclusive as elements supporting both can be found in our data.

Bertola et al. (2014b) suggested the total number of switches during a category fluency test could be used as a proxy for measuring executive function and found that the total number of switches decreases with impaired cognition. Our work suggests that this approach does not fully explore semantic recall and that including timing in the analysis reveals more when attempting to understand an individual’s ability to switch between categories.

Our data suggest that, before detrending, lower performance on semantic recall tests is not associated with an increased amount of time required to switch between semantic categories since LP intercluster time was not significantly different from MP or HP (Figure 3A). The raw data instead suggest that impaired performance on category fluency tests is associated with longer intracluster response times (Figure 3B), and the reduced number of switches produced by LP (Figure 3C) is attributable to the longer intracluster times.

This analysis of our raw data supports the structure-loss model which attributes word-finding deficits to a breakdown in the associative networks underlying semantic memory, i.e., individuals with difficulty making connections between semantically related words have an increased mean intracluster response time, as we observed. The retrieval-slowing model, by contrast, attributes measured deficits in semantic fluency to a more widespread general slowing of retrieval processes while the underlying semantic structure is preserved. The retrieval-slowing model predicts that both intracluster and intercluster response times would increase with worsening performance on category fluency tests. Without detrending, our raw data do not support the retrieval-slowing model because significant differences were seen only in intracluster times, but not intercluster times.

The story becomes more complex when considering the detrended data, where effects due to general retrieval-slowing are revealed. In contrast to the raw data results, LP are found to spend significantly less time between clusters than MP or HP (Figure 4A, *p* < 0.05 and 0.01, respectively), and there are no differences among the groups in detrended intracluster response times (Figure 4B). Detrending the intercluster switch times distinguishes the LP group from the others: it reveals that the time between clusters becomes shorter compared to the other groups (Figure 4A), suggesting that semantic access is not impaired. Detrending the data alters the clustering and switching patterns apparent in the raw data, and hence supports the retrieval-slowing model; removing the effects of exponential slowing altered the patterns of temporal dynamics across the three performance groups. These detrended results together with the raw data suggest that neither retrieval-slowing nor semantic access alone can completely explain word retrieval deficits seen in LP, and indeed these hypotheses need not be mutually exclusive.

One explanation suggested by our data for the faster detrended intercluster switch times by LP is that LP individuals may name the first animal that comes to mind without considering how many other animals “related” to that one they can name. The less time one may take to decide on an animal group, the faster one will switch to a new group, but ultimately fewer animals in the new category are quickly accessed. That is to say, switching ability is related to executive control, which may be impaired in individuals with dementia (Carter et al., 2012; Kirova et al., 2015). By contrast, higher performing individuals may purposefully take more time when switching between categories in order to decide on a “next best” category. They may “strategically” select a category that will allow access to a greater number of animal names, thus increasing the number of responses for the time they spend in a cluster, which is the converse of what happens with lower performing individuals. The less time one takes to decide on an animal group, the faster one will switch to a new group, but ultimately fewer animals in the new category are quickly accessed. The difference in intercluster response times may be undetectable prior to detrending because the “conscious” slowing between categories by HP is indistinguishable from the general retrieval-slowing of LP when measuring raw intercluster response times.

Beyond this empirical reasoning, several lines of research have suggested mechanisms governing performance beyond what is presented in this article. It is known that choice of a smaller category such as “polar animals” will result in fewer items named than larger categories such as “animals”. Additionally, the Weibull distribution model suggests that a “first past the gate” competition is operative (Meyer et al., 2012). Another model for analyzing the temporal structure of category fluency responses is that of a “coalescent stochastic process model” (Queau et al., 2015). Semantic “space” may also be conceived as a hierarchical network of associations, so that common animals (e.g., cat or dog) have more associations, providing a rationale for why they tend to be named earlier. It should be noted in this context that both the structure loss hypothesis and the slowed retrieval process may be operative as well (Rohrer et al., 1995).

While the raw data might support the structure-loss model because LP takes longer to name the same number of animals in a cluster (Figures 3B,D), the detrended data show an effect attributable to the retrieval-slowing model. Regardless of the mechanism responsible for this outcome (which is likely multifactorial and includes retrieval-slowing, structure-loss, and impaired executive function among others), the raw intercluster response times and the detrended intercluster response times both stand in contrast to the notion that impaired semantic fluency is associated with an inability to switch between semantic clusters quickly, as previously measured by switch number.

One limitation of this study is the assignment of tertile cutoffs for the three groups. Though a number of instruments are available clinically to help diagnose severity of cognitive impairment (e.g., MMSE and SLUMS were used in the larger CEPRO study), we elected to divide our subjects into groups based solely on semantic fluency performance. This did not provide us with clinically derived cutoff values to distinguish the groups cognitively, but avoided skewing the groups based on other cognitive deficits (e.g., visuospatial, abstraction, orientation) that affect outcomes in the clinical instruments. Group assignments directly reflect the key metric we investigated. Other limitations of our study include a relatively small sample size and the nature of a cross sectional study that does not allow for tracking the effects of the aging process on our outcome measures over time.

As long as the treatment mainstay of AD and related dementias consists of acetylcholinesterase inhibitors and therapies that work by slowing the apparent progression of disease, early diagnosis and identification of those at high risk will remain critical (Chong and Sahadevan, 2005; Levey et al., 2006; Monsell et al., 2014). Neuropsychological assessments such as semantic fluency testing assist in identifying individuals with MCI or preclinical AD who are otherwise asymptomatic (Leibing, 2014), and so are essential to early identification. The utility of continuous time measurement is that it adds a new dimension to the analyses of semantic fluency testing, providing a more granular analysis of the dynamics of word production and clustering and switching compared to previous binning procedures (Meyer et al., 2012). Further, detrending allows for the study of specific neural networks underlying semantic fluency by removing the confounding effects of cognitive impairment on retrieval speed. Better understanding of these mechanisms may lead to improved detection of preclinical dementia. Developing these and other methods to detect the subtler deficits of cognition are essential to maximizing the effectiveness of current treatments.

## Author Contributions

SL, FML, AJL and WAW: analyzed the data. SL, FML, AJL, KAS, MS and CT: contributed to writing the manuscript.

## Funding

“Assessing Early Alzheimer and At-Risk Groups with Patient Reported Outcomes” was supported by ARRA grant AG038825-01 from the National Institute on Aging to KAS and MS. The funders had no role in study design, data collection and analysis, decision to publish, or preparation of the manuscript.

## Conflict of Interest Statement

The authors declare that the research was conducted in the absence of any commercial or financial relationships that could be construed as a potential conflict of interest.
